# Crystal structures of a llama VHH antibody BCD090-M2 targeting human ErbB3 receptor

**DOI:** 10.12688/f1000research.13612.2

**Published:** 2018-07-04

**Authors:** Igor E. Eliseev, Anna N. Yudenko, Vera V. Vysochinskaya, Anna A. Svirina, Anna V. Evstratyeva, Maria S. Drozhzhachih, Elena A. Krendeleva, Anna K. Vladimirova, Timofey A. Nemankin, Viktoria M. Ekimova, Andrey B. Ulitin, Maria I. Lomovskaya, Pavel A. Yakovlev, Anton S. Bukatin, Nickolay A. Knyazev, Fedor V. Moiseenko, Oleg B. Chakchir

**Affiliations:** 1 St. Petersburg National Research Academic University RAS, St. Petersburg, 194021, Russian Federation; 2 CJSC Biocad, St. Petersburg, 198515, Russian Federation

**Keywords:** cancer, therapeutic antibodies, receptor tyrosine kinase, HER3, single-domain antibody, nanobody, crystal structure

## Abstract

**Background**: The ability of ErbB3 receptor to functionally complement ErbB1-2 and induce tumor resistance to their inhibitors makes it a unique target in cancer therapy by monoclonal antibodies. Here we report the expression, purification and structural analysis of a new anti-ErbB3 single-chain antibody.

**Methods**: The VHH fragment of the antibody was expressed in *E. coli SHuffle* cells as a SUMO fusion, cleaved by TEV protease and purified to homogeneity. Binding to the extracellular domain of ErbB3 was studied by surface plasmon resonance. For structural studies, the antibody was crystallized by hanging-drop vapor diffusion in two different forms.

**Results**: We developed a robust and efficient system for recombinant expression of single-domain antibodies. The purified antibody was functional and bound ErbB3 with K *_D_*=15±1 nM. The crystal structures of the VHH antibody in space groups C2 and P1 were solved by molecular replacement at 1.6 and 1.9 Å resolution. The high-quality electron density maps allowed us to build precise atomic models of the antibody and the putative paratope. Surprisingly, the CDR H2 existed in multiple distant conformations in different crystal forms, while the more complex CDR H3 had a low structural variability. The structures were deposited under PDB entry codes 6EZW and 6F0D.

**Conclusions**: Our results may facilitate further mechanistic studies of ErbB3 inhibition by single-chain antibodies. Besides, the solved structures will contribute to datasets required to develop new computational methods for antibody modeling and design.

## Introduction

Receptor tyrosine kinases ErbB1-4 (HER1-4) receive inputs from growth factors and transmit signals to the cell nucleus, thus regulating key cellular processes such as growth, differentiation, migration, and apoptosis ^1^. Aberrations of ErbB signaling, caused by mutations or receptor overexpression, are associated with the development of a wide variety of cancers. The essential role of ErbB receptors in tumor development makes them a unique target in cancer therapy by monoclonal antibodies ^2^. Therapeutic antibodies often act on the first stage of signal transduction by inhibiting ligand binding or receptor dimerization.

The first two members of the family, ErbB1 (EGFR, HER1) and ErbB2 (HER2/neu), were early recognized as promising drug targets because of their frequent overexpression in tumors. The examples of successful application of anti-ErbB antibodies in cancer treatment include *cetuximab* (anti-EGFR) in head and neck cancer therapy and *trastuzumab* (anti-HER2) in breast cancer treatment. The role of the third member, ErbB3, has long been underestimated because it lacks intrinsic tyrosine kinase activity. However, its ability to form functional dimers with ErbB1-2 and to confer resistance to their inhibitors makes ErbB3 an important drug target ^3^. Particularly, it was shown that inhibition of ErbB2 with *lapatinib* caused transcriptional up-regulation of ErbB3, which was then phosphorylated by residual ErbB2 kinase activity thus limiting antitumor effect ^4^. A comprehensive clinical study revealed that ErbB3 overexpression was a significant marker of reduced survival in patients with breast cancer ^5^.

This new data stimulated the development of anti-ErbB3 antibodies, which are at various stages of clinical trials ^6^. The rational antibody design requires knowledge of molecular mechanism of ErbB3 inhibition. Recently, several structures of ErbB3-antibody complexes were solved ^7– 9^. Surprisingly, these structures showed that antibodies target entirely different epitopes on the receptor: extracellular domain I ^7^, domains II and IV ^8^, or domain III ^9^.

In Russia, anti-ErbB3 antibodies are developed by BIOCAD biotechnology company. The phage display selection of antibodies from immunized llamas and subsequent sequencing allowed the identification of several anti-ErbB3 single-chain antibodies. As a part of our ongoing effort to elucidate the molecular mechanism of ErbB3 inhibition and ultimately open up a possibility of therapeutic application of these antibodies, we study their thermodynamic stability ^10^, functional properties, and structure. In this work, we describe the expression, purification, crystallization and structural analysis of the variable fragment of an antibody BCD090-M2, which demonstrated an affinity to the extracellular domain of ErbB3 in preliminary experiments.

## Methods

### Plasmid construction

Gene fragment encoding the VHH fragment of the antibody was cloned into pSolSUMO expression vector (Lucigen), following the manufacturer’s recommendations. Briefly, the fragment was PCR amplified using the primers: forward 5’-aatctgtacttccagggtcaggtgcagctggtgcag-3’, reverse 5’-gtggcggccgctctattatgaggagacggtgaccgt-3’, with the first 18 nucleotides in both primers matching the ends of linearized pSolSUMO vector. Following the amplification, the fragment was mixed with linearized pSolSUMO vector and used to transform chemically competent *E. cloni 10G* cells (Lucigen). The resultant plasmid pSolSUMO-BCD090-M2, encoding SUMO with N-terminal hexahistidine tag fused to BCD090-M2 through a TEV recognition site, was sequenced and used for further protein expression in *E. coli SHuffle T7 Express* cells (NEB).

### Protein expression and purification

Chemically competent *E. coli SHuffle T7 Express* cells (NEB) were transformed by pSolSUMO-BCD090-M2, and single colonies were used to start small-scale overnight cultures. Then 2–4 l bacterial cultures were inoculated by 1:100 volume of overnight culture and grown in 2xYT supplemented with 50 *µ*g/ml kanamycin at 37°C. At OD 0.6–0.8, protein expression was induced by the addition of L-Rhamnose to a final concentration 5 mM, temperature was lowered to 27–29°C and cells were grown overnight for additional 14–15 h. Cells were then harvested by centrifugation at 10000g (5 min), resuspended in IMAC buffer (50 mM Na _2_HPO _4_ pH 8.0, 0.3 M NaCl, 5 mM Imidazole) with 1 mM PMSF, 0.5 mM EDTA as protease inhibitors and lysed by ultrasonication. Cell debris were pelleted by centrifugation at 40000g (20 min), and the cell extract supernatant was filtered through 0.22 *µ*m membrane. The solution was loaded on 1 ml IMAC column cOmplete (Roche) at 0.5–1 ml/min, the column was washed with 20–40 column volumes of IMAC buffer, and then the protein was eluted by IMAC buffer with 0.3 M Imidazole.

After elution from IMAC column, sample purity was usually higher than 90% as judged by SDS-PAGE, and the protein was cleaved by TEV protease. Sample was first dialyzed against TEV buffer (30 mM Tris pH 8.0, 0.5 mM EDTA, 1 mM DTT) overnight at 4°C, then mixed with TEV protease at 1:40 to 1:80 enzyme to substrate ratio and cleaved for 4 h at 25°C with mild agitation. Histidine-tagged SUMO was then removed by three repeats of negative IMAC chromatography in batch mode. Sample in TEV buffer with 50 mM NaCl and 5 mM Imidazole was mixed with 200 *µ*l Ni-NTA agarose resin (Qiagen) and incubated for 30 mins with mild agitation, the agarose beads were pelleted by a short centrifugation, and the supernatant was taken and used for the next round of SUMO depletion.

Finally, the cleaved VHH antibody BCD090-M2 was purified by an additional polishing step of high-resolution cation exchange chromatography on a MonoS 5/50 GL column (GE Healthcare). The protein was dialyzed against IEX buffer (20 mM Na Acetate pH 6.0) overnight at 4°C, loaded on a pre-equilibrated column, and eluted by IEX buffer with 0–0.5 M NaCl gradient over 20 column volumes. The peak fractions analyzed by SDS-PAGE were pooled, dialyzed overnight against Sample buffer (20 mM HEPES pH 7.5, 50 mM NaCl), and concentrated on a 10 kDa Amicon centrifuge concentrators (Millipore). Protein concentration was measured spectrophotometrically with the parameters *ε*=27055 M ^−1^cm ^−1^, MW=13955 Da calculated from the amino acid sequence with the ProtParam tool ^11^.

For affinity measurements, we produced an extracellular domain (residues 21–643) of the human ErbB3 receptor using a pEE vector with a CMV promoter carrying the ErbB3 gene fragment followed by a hexahistidine tag and a FLAG-tag. CHO-T-HC cells were transfected with PEI and grown one day in HyCell TransFx-C media (GE Healthcare) at 37°C. On the day 2, the temperature was lowered to 32°C, and cells were grown for an additional 8 days. Then the cells were harvested by sterile filtration through Opticap XL capsule filters (Millipore), the clarified culture fluid was supplemented with 1 mM NiCl _2_ and 10 mM Imidazole, and loaded on a HisTrap HP (GE Healthcare) column equilibrated with IMAC buffer. The column was washed with 10 volumes of IMAC buffer, and the protein was eluted with 0.3 M Imidazole. After elution from IMAC column, the protein was further purified by size-exclusion chromatography on HiLoad 16/600 Superdex 200pg (GE Healthcare), dialyzed against PBS and concentrated on a 10 kDa Amicon centrifuge concentrators (Millipore).

### Affinity measurement

Interaction of the recombinantly expressed VHH antibody BCD090-M2 with the extracellular domain of the ErbB3 receptor was studied by surface plasmon resonance technique using a Biacore T200 instrument (GE Healthcare). The purified extracellular domain was diluted in 10 mM Na Acetate buffer pH 4.5 to a final concentration of 100 *µ*g/ml and immobilized on CM5 chip via amine coupling with EDC/NHS, following the manufacturer’s recommendations. The BCD090-M2 stock solution was serially diluted in HBS buffer supplemented with BSA (10 mM HEPES pH 7.4, 0.15 M NaCl, 50 *µ*g/ml BSA) to prepare concentration series from 1 *µ*M down to 0.49 nM. Each sample was injected to a cell with immobilized receptor and a reference cell at 10 *µ*l/min flow rate and association/dissociation time of 30 min. All measurements were performed at 37°C. Between the samples the chip surface was equilibrated with HBS for 10 min without an additional regeneration step. The sensograms were reference-subtracted and analyzed in Biacore T200 evaluation software. The equilibrium dissociation constant was obtained by fitting the response measured at 5 s before the end of association phase as a function of analyte concentration. The reported value was a mean calculated from three experiments.

### Crystallization

The BCD090-M2 crystallization conditions were screened using the commercial sparse-matrix screens Classics I and II, AmSO _4_ (Qiagen), Clear Strategy I and II, Morpheus (Molecular Dimensions). Crystallization experiments were set up by sitting-drop vapor diffusion method in 96-well plates at 19°C. Each crystallization drop consisted of 100 nl protein solution at a concentration of 17 mg/ml in Sample buffer (20 mM HEPES pH 7.5, 50 mM NaCl) and 100 nl reservoir solution. The screening revealed the two classes of promising conditions, one with salts of carboxylic acids and PEG (Morpheus #73, #76), and the other with divalent cations and PEG (Classics II #64). The crystallization experiments with the identified conditions were reproduced in 24-well Linbro plates by hanging-drop vapor diffusion method with 2 *µ*l drop volume, 1:1 ratio, and 0.5 ml reservoir volume at 20°C. In the case of first crystallization condition (Morpheus #73), well-formed crystals of 0.2–0.3 mm size appeared after 3–4 days in the hanging-drop experiments. Preliminary X-ray experiments showed diffraction up to 1.6 Å, therefore no further optimization was attempted; the crystals for data collection were obtained using the original precipitant solution #73 from Morpheus screen. In the case of the second crystallization condition (Classics II #64), optimization experiments were made to increase crystal size and improve morphology. It appeared that among divalent cations only Cd ^2+^ was essential for crystallization, the optimized reservoir solution had the following composition: 0.1 M MES pH 6.5, 12% PEG 3350, 5 mM CdSO _4_. Large crystals up to 0.7 mm usually appeared after 4–5 days and diffracted below 2.0 Å.

### Data collection and processing

The crystals grown in the first crystallization condition (Morpheus #73) were mounted in loops, cryoprotected in the mother liquor with 25% glycerol, and flash cooled in cold nitrogen gas stream. The crystals grown with Cd ^2+^ deteriorated upon soaking in different cryoprotectant solutions, and so were mounted in thin-walled quartz capillaries (Hampton Research) for room-temperature data collection. All diffraction data were collected on a Kappa Apex II diffractometer (Bruker AXS) using CuK *α* radiation. The datasets were integrated with SAINT V8.18C and scaled with SADABS v. 2008/1 software ^12^. The crystal grown in the first condition diffracted to 1.6 Å, the unit cell parameters were a=65.76 Å, b=38.93 Å, c=47.48 Å, *α*= *γ*=90°, *β* =102.24°, the space group C2 was determined with XPREP v. 2008/2 ^12^. Notably, the crystal grown in the second condition with Cd ^2+^ appeared triclinic with unit cell parameters a=35.77 Å, b=41.53 Å, c=46.49 Å, *α*=89.99°, *β* =67.92°, *γ*=76.06° and two copies of the VHH antibody in the asymmetric unit, and diffracted to a slightly lower resolution of 1.9 Å. The details of data collection and processing are summarized in Table 1.

**Table 1.** Data collection and refinement statistics. Statistics for the highest-resolution shell are shown in parentheses.

Dataset name PDB entry code	6EZW	6F0D
Diffraction source	Kappa Apex II (Bruker AXS)	Kappa Apex II (Bruker AXS)
Detector	Apex II CCD	Apex II CCD
Temperature, K	100.0	293.15
Distance, mm	38	38
Image width, °	1.0	1.0
Images	599	2613
Wavelength, Å	1.54184	1.54184
Resolution range, Å	33.3–1.598 (1.656–1.598)	32.34–1.9 (1.968–1.9)
Space group	C 1 2 1	P 1
Unit cell: a b c, Å, *α β γ*, *◦*	65.76 38.93 47.48 90 102.24 90	35.77 41.53 46.49 89.99 67.92 76.06
Total reflections	80881 (3994)	246903 (9578)
unique	15511 (1365)	18859 (1885)
Multiplicity	5.2 (2.9)	13.1 (5.1)
Completeness, %	96.50 (81.41)	96.75 (91.94)
Mean I/ *σ*(I)	13.89 (2.06)	14.86 (2.26)
Wilson B-factor	9.36	15.28
R-merge	0.073 (0.446)	0.144 (0.806)
R-meas	0.081 (0.539)	0.15 (0.906)
R-pim	0.033 (0.296)	0.040 (0.406)
CC1/2	0.998 (0.794)	0.997 (0.412)
CC*	0.999 (0.941)	0.999 (0.764)
Reflections used in refinement	15157 (1266)	18248 (1733)
Reflections used for R-free	1517 (126)	1829 (174)
R-work	0.182 (0.28)	0.174 (0.257)
R-free	0.213 (0.288)	0.218 (0.322)
CC(work)	0.964 (0.810)	0.971 (0.828)
CC(free)	0.945 (0.778)	0.951 (0.674)
Number of non-hydrogen atoms	1171	2119
protein	981	1962
ligands	0	2
solvent	190	155
Protein residues	128	256
RMS (bonds)	0.006	0.014
RMS (angles)	0.82	1.53
Ramachandran		
favored, %	98.41	97.22
allowed, %	1.59	2.78
outliers, %	0.00	0.00
Rotamer outliers, %	0.78	1.96
Clashscore	8.89	5.76
Avg. B-factor	14.36	19.86
protein	12.20	19.29
ligands	–	15.44
solvent	25.49	27.19

## Results and discussion

**Figure 1.**
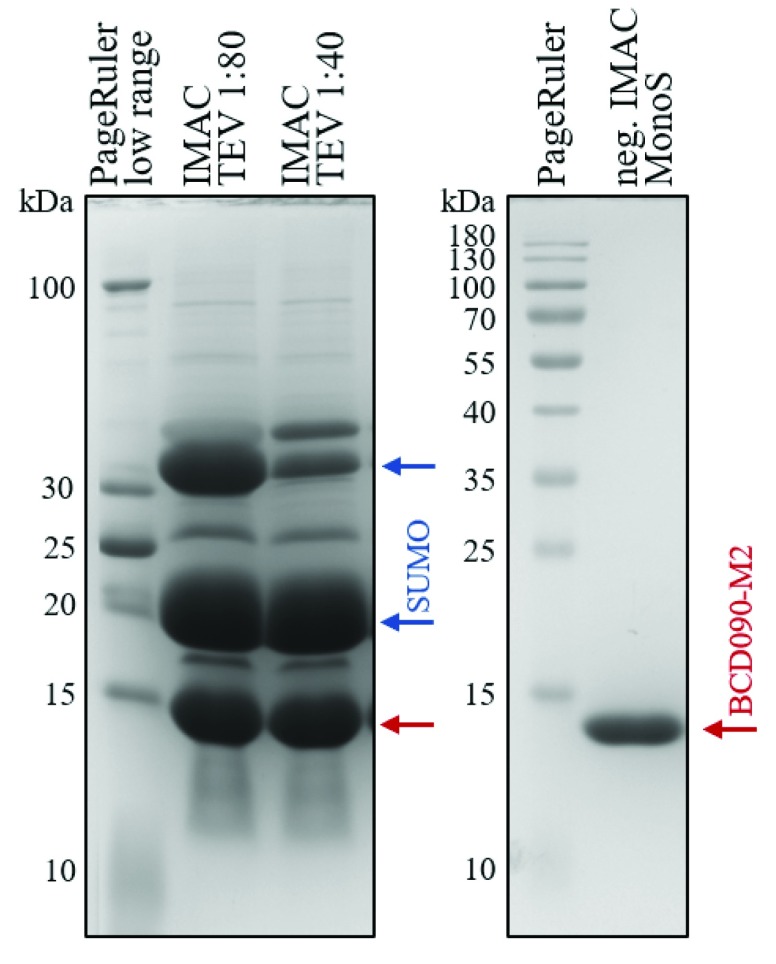
Expression and purification of BCD090-M2 VHH antibody. The antibody was expressed in the cytoplasm of *E. coli* SHuffle cells as His _6_-SUMO fusion, purified by IMAC and cleaved with TEV protease at 1:80 or 1:40 enzyme:substrate ratio. The bottom band marked with a red arrow corresponds to BCD090-M2, the middle band marked with a blue arrow is a histidine-tagged SUMO, and a top band is an uncleaved protein. After the cleavage, BCD090-M2 was further purified by negative IMAC and cation-exchange chromatography on MonoS, the SDS-PAGE analysis of the purified antibody is presented in the right panel.

The llama VHH antibody BCD090-M2 was expressed in soluble form in the cytoplasm of *E. coli SHuffle T7 Express* cells as a SUMO fusion. The *SHuffle* strain ^13^ has deletions of the genes *trxB* and *gor*, and constitutively expresses a chromosomal copy of the disulfide bond isomerase DsbC to promote formation of correct disulfide bonds in recombinant proteins. The system proved very efficient for single-chain antibody expression, the typical protein yield in our experiments was 50 mg of a fusion protein per liter of a bacterial culture after the first IMAC step. For the IMAC we used new chromatography media cOmplete (Roche), which withstands high EDTA and DTT concentrations and demonstrates a different binding strength and specificity compared to traditional Ni-NTA resins. Particularly, our histidine-tagged fusion protein eluted at 30 mM Imidazole concentration in gradient elution experiments, and addition of only 5 mM Imidazole to cell extract and wash buffer efficiently suppressed non-specific binding, resulting in greater than 90% purity after the first chromatography step. The protein was then cleaved by TEV protease to obtain untagged antibody BCD090-M2 with nearly native N-terminus, differing from the original sequence by a single additional glycine residue left from the TEV recognition site. The extent of cleavage was monitored by SDS-PAGE and typically was more than 70% at 1:80 enzyme ratio and more than 85% at 1:40 ratio, as shown in Figure 1 (left panel).

The bottom band with apparent molecular mass 14 kDa corresponds to processed BCD090-M2, the middle band corresponds to His _6_-SUMO (13 kDa) and migrates anomalously slow probably due to positively charged histidine tag, and the top band is the uncleaved protein. The processed BCD090-M2 was separated from His _6_-SUMO and intact fusion protein by negative IMAC chromatography in batch regime, and then polished by an additional step of cation exchange chromatography on a MonoS column. After elution from MonoS, the VHH antibody was almost pure as judged by SDS-PAGE shown in Figure 1 (right panel).

**Figure 2.**
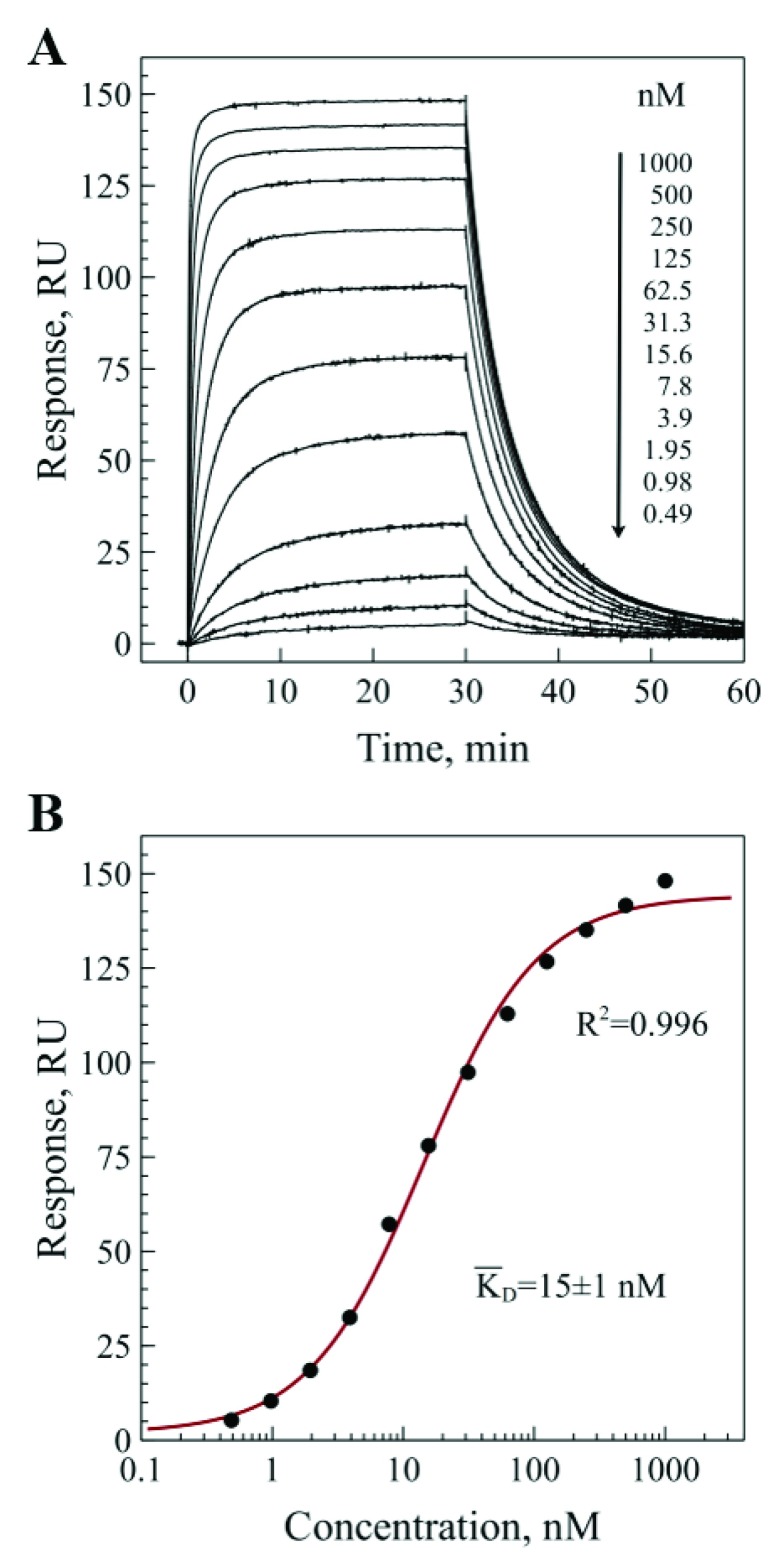
Binding of BCD090-M2 VHH antibody to the extracellular domain of human ErbB3 receptor analyzed by surface plasmon resonance. The purified extracellular domain (residues 21-643) was immobilized on CM5 chip surface. **A**) binding sensograms measured for different concentrations of BCD090-M2; **B**) fitting of the measured response as a function of concentration of free BCD090-M2 in solution with a simple bimolecular equilibrium binding model and averaging over three experiments gives *K_D_*=15±1 nM.

The surface plasmon resonance experiment confirmed that the recombinant VHH antibody was functional and efficiently bound to the immobilized receptor. The representative experimental binding sensograms are shown in Figure 2A, and the processed data fitted with an equilibrium binding model is shown in Figure 2B. The experiments showed little variation and yielded mean equilibrium dissociation constant for monovalent binding *K_D_*=15±1 nM. Although the monovalent affinity is a fundamental characteristic of antibody-antigen interaction, the avidity of a full-length bivalent antibody can be much higher. Typically, the enhancement of avidity due to a bivalent interaction is 3–4 orders of magnitude and depends strongly on the surface concentration of antigen ^14^.

The protein was successfully crystallized in two different forms: in space group C2 with a single copy of BCD090-M2 in the asymmetric unit and in P1 with two molecules and two cadmium ions in the unit cell. After data collection and processing, all further data analysis procedures, including phasing, model building, and refinement, were conducted in Phenix software suite v. 1.11.1_2575 ^15^. To solve the structures by molecular replacement, we selected a set of single-domain antibodies from PDB with the highest homology with BCD090-M2. We then processed the search models with Sculptor ^16^ to delete residues that were not aligned with the target and to prune sidechains by the Schwarzenbacher algorithm ^17^, and performed molecular replacement using Phaser v. 2.7.16 ^18^. The best molecular replacement solutions for both datasets were obtained with a nanobody targeting complement receptor Vsig4, PDB:5IMK ^19^. Then we used phenix.autobuild ^20^ to automatically build the framework regions of the BCD090-M2 and Coot v. 0.8.6.1 ^21^ to manually fit the missing CDRs into the experimental electron density. The structures were refined using phenix.refine ^22^ (see Table 1 for refinement statistics) and deposited to the Protein Data Bank under entry codes 6EZW and 6F0D. All figures were generated using PyMOL.

**Figure 3.**
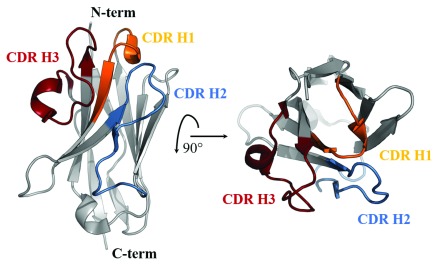
Ribbon diagram of the crystal structure of BCD090-M2 in space group C2 at 1.6 Å resolution (PDB:6EZW). Framework regions are grey and CDRs are colored.

**Figure 4.**
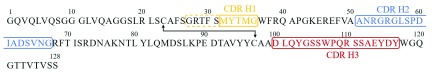
Amino acid sequence of BCD090-M2. The numbering is the same as in the PDB files 6EZW and 6F0D. The CDR regions according to Kabat definition are indicated with color and frames. In the case of CDR H1, the AbM definition (dashed frame, residues 27-36) was used.

The overall structure of BCD090-M2 crystallized in space group C2 is shown in Figure 3. The framework regions are drawn as grey ribbons, and the CDRs are colored in orange (H1), blue (H2), and red (H3). The CDRs were defined according to Kabat ^23^ as shown in Figure 4.

For the CDR H1, we also used AbM definition, a combination of Kabat and Chotia ^24^ definitions, which better matches the loop in protein structure. The high resolution of the dataset and good quality of the electron density maps allowed us to build a precise atomic model of the antibody. We then used a *PyIgClassify* ^25^ to analyze the structures of BCD090-M2 CDRs using a comprehensive database of antibody CDR loop conformations. The CDR H1 and H2 belonged to two large common clusters, H1-13-1 and H2-10-2 respectively. The CDR H3 was not assigned to any known structure cluster, probably due to size (18 residues) and complex structure of the loop, which has a cis-proline and four aromatic residues. It is consistent with an observation that CDR H3 loops are very diverse in structure and a few clusters of significant size are present in the database.

**Figure 5.**
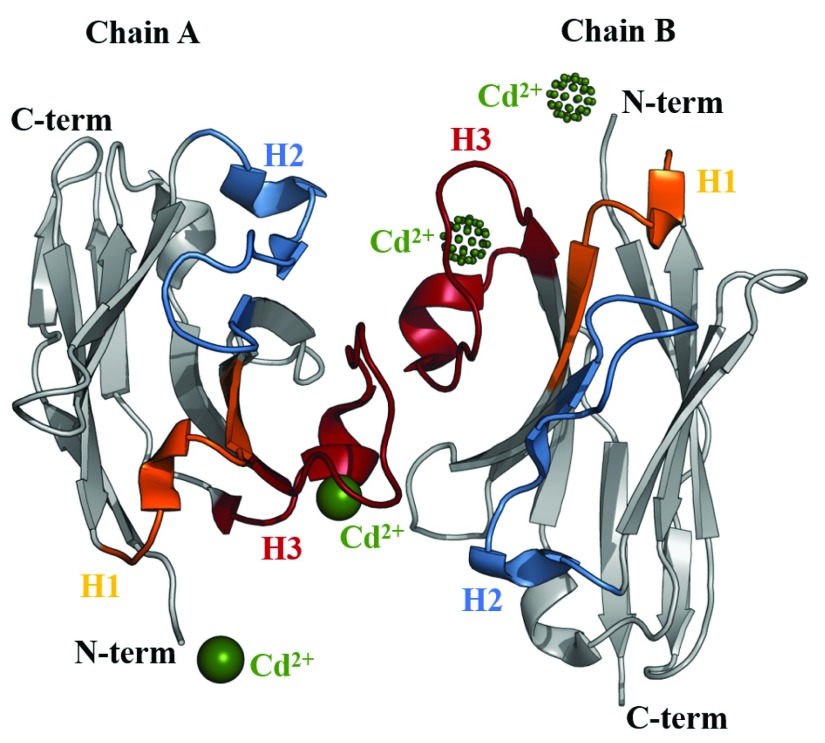
Ribbon diagram of the crystal structure of BCD090-M2 in space group P1 at 1.9 Å resolution (PDB:6F0D). Asymmetric unit contains two copies of the VHH antibody and two cadmium ions which are pictured as green spheres, two symmetry-related ions from the neighbor unit cell are shown in dots. Framework regions are grey and CDRs are colored.

The structure of the BCD090-M2 dimer crystallized in space group P1 with cadmium ions is shown in Figure 5. Two cadmium ions in the unit cell are pictured as green spheres, and two symmetry-related ions from the neighbor unit cell are shown in dots. Each cadmium ion is bound by residues Asp100, Glu114, and Asp116 belonging to CDR H3, and by N-terminal glycine residue of an antibody molecule from neighbor unit cell. Thus, intermolecular interaction through cadmium ions effectively define the lattice of a crystal. This finding is consistent with a previous general observation that cadmium can induce the formation of protein crystals or improve their quality ^26^.

**Figure 6.**
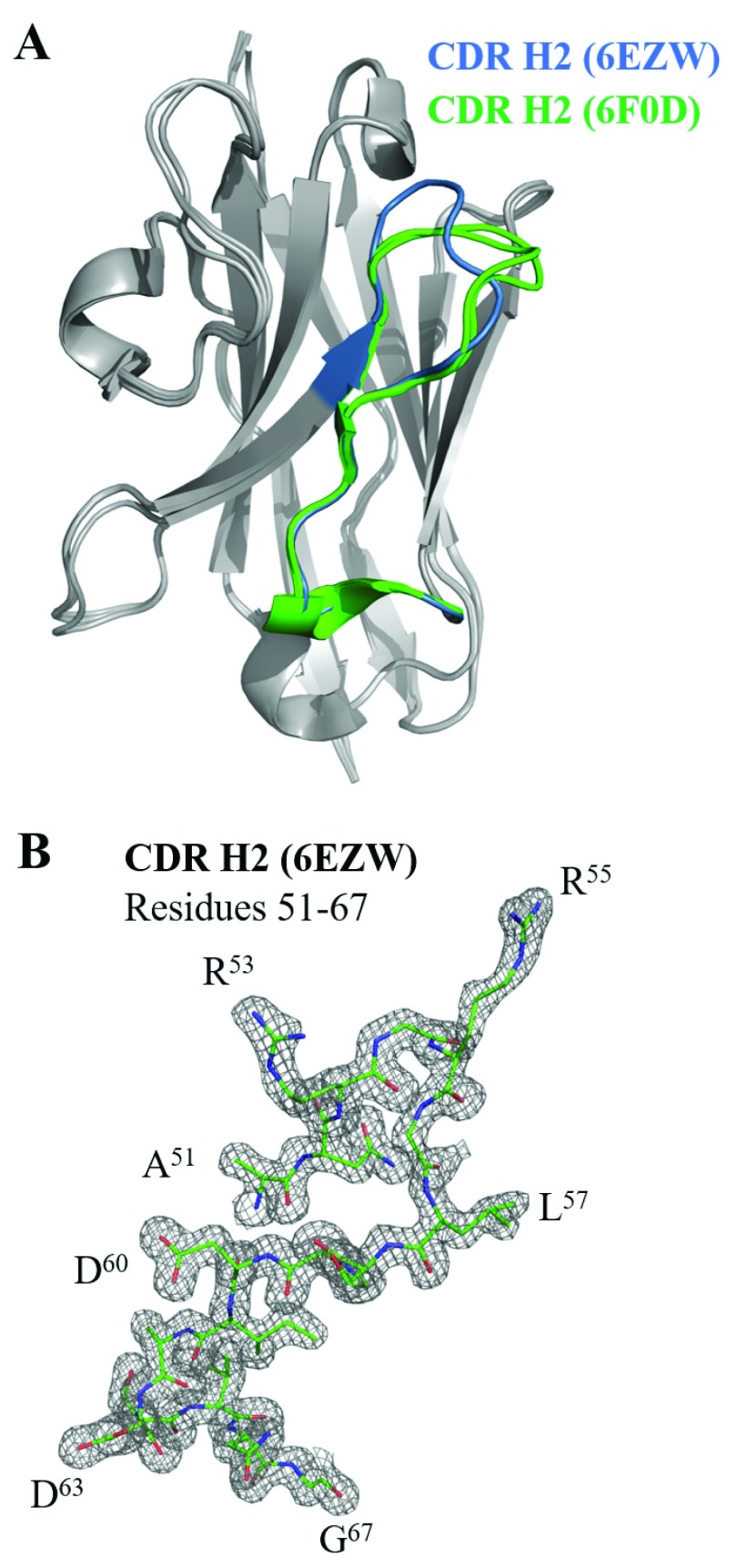
Structural comparison of BCD090-M2 crystallized in different space groups. **A**) the three structures, 6EZW, 6F0D chain A, and 6F0D chain B, were superimposed, the structural alignment was based on C ^*α*^ atoms of framework regions. CDRs H1 and H3 showed little structural variation, while CDR H2 adopted different conformations in 6EZW and 6F0D, which are shown in blue and green color. **B**) Electron density map (F-obs, Phi-model, 1.0 *σ*) for CDR H2 (residues 51-67) of 6EZW. The quality of electron density maps allowed us to unambiguously trace the loop and place amino-acid side chains.

Finally, we analyzed the structural variability of the overall antibody fold and the putative paratope by comparing the three solved structures 6EZW, 6F0D (A), and 6F0D (B). We first performed the structural alignment based on C ^α^ atoms of the framework regions, which is shown in Figure 6A.

The overall fold of the antibody had a very low structural variability, the RMSD between framework C ^α^ atoms in 6EZW and 6F0D was 0.19 Å. The conformational mobility of the short CDR H1 was also low, with C ^α^ RMSD between 6EZW and 6F0D 0.28 and 0.31 Å for chains A and B, respectively. Surprisingly, the most complex CDR H3 had a rigid conformation, which was not significantly altered by binding of a cadmium ion and intermolecular interaction in the asymmetric unit of 6F0D. The C ^α^ RMSD for CDR H3 between two structures was 0.61 (chain A) and 0.57 Å (chain B). The largest structural variation was observed in the loop H2, as seen in Figure 6A. The C ^α^ RMSD was 1.81 and 1.77 Å for chains A and B, respectively, and the largest difference was observed in the region Arg53-Leu57. Despite the probable flexibility of this loop, in both crystals it was well-resolved in the electron density maps, as shown in Figure 6B, allowing us to unambiguously place all residues. As a result of the observed structural change, the CDRs H2 in 6F0D were attributed to a different from 6EZW distant structural clusters H2-10-6 (chain A) or H2-10-7 (chain B) by *PyIgClassify* ^25^.

Uncropped gel from Figure 1 and raw output data from Biacore software http://dx.doi.org/10.5256/f1000research.13612.d209631
Copyright: © 2018 Eliseev IE et al. 2018 Data associated with the article are available under the terms of the Creative Commons Zero "No rights reserved" data waiver (CC0 1.0 Public domain dedication).

## Conclusions

In conclusion, here were present expression and purification, functional and structural analysis of a new single-domain llama antibody against human ErbB3. We crystallized the antibody in two different forms and solved high-resolution structures, giving an insight to the organization of the putative ErbB3 binding paratope. We believe this data may facilitate further studies of mechanisms of ErbB3 inhibition by single-chain antibodies. Besides, the solved structures will contribute to datasets that are required to develop new robust computational methods for antibody modeling and design.

## Data availability

The data referenced by this article are under copyright with the following copyright statement: Copyright: © 2018 Eliseev IE et al.

Data associated with the article are available under the terms of the Creative Commons Zero "No rights reserved" data waiver (CC0 1.0 Public domain dedication). 

The atomic coordinates and structure factors can be accessed under PDB codes 6EZW and 6F0D.

Dataset 1: Uncropped gel from Figure 1 and raw output data from Biacore software. DOI, 10.5256/f1000research.13612.d209631 ^27^

The plasmids and recombinant proteins used in this study are available from Igor Eliseev (corresponding author) upon request.
