# Supplementary material for: Crystal structures of a llama VHH antibody BCD090-M2 targeting human ErbB3 receptor
Source: F1000Res. 2018 Jul 4;7:57. Originally published 2018 Jan 16. [Version 2] doi: 10.12688/f1000research.13612.2 (PMC6097396; doi:10.12688/f1000research.13612.2)
Supplement: Peer review history

# Peer review: Referee response for version 2

**DOI:** 10.5256/f1000research.16763.r35737

**Published:** 2018-08-15

**Authors:** Kathryn M. Ferguson

**Affiliations:** 1 Department of Physiology, Perelman School of Medicine, Philadelphia, PA, USA

The SPR data in this revised manuscript are very much improved.

I have read this submission. I believe that I have an appropriate level of expertise to confirm that it is of an acceptable scientific standard.

# Peer review: Referee response for version 1

**DOI:** 10.5256/f1000research.14786.r32141

**Published:** 2018-04-10

**Authors:** Kathryn M. Ferguson

**Affiliations:** 1 Department of Physiology, Perelman School of Medicine, Philadelphia, PA, USA

The authors purify a VHH fragment corresponding to an camelid single-chain antibody previously selected from a Llama derived phage antibody library. They measure binding of this VHH (BCD090-M2) to the extracellular region of ErbB3 that was immobilized to a CM5 biosenor chip, using Surface plasmon resonance (SPR). An apparent K _D_ of 1μM is reported. The authors solve the X-ray crystal structure of the VHH in two different crystal forms and observe differences in the conformation of CDR H2. 

 The SPR sensograms do not come to a plateau during each injection, which is most obvious at the higher concentrations, so it is not a saturation binding response for each concentration. The Response data that are plotted in Figure 2B for each concentration of VHH do not correspond to the response values 5 seconds before the end of the injection in the sensograms plotted in Figure 2A. It looks more like the values 50 seconds before end of injection. There is something wrong here. Perhaps the data in A and B are from two different experiments? No error is given on the K _D_ value so it is not clear if these data were repeated. I also worry about use of regeneration using glycine at pH 2. It is highly likely that this will denature the immobilized ErbB3. These data are thus questionable. No information on binding for the intact antibody is referenced or provided. The VHH is clearly folded. The question is whether it really binds to ErbB3. 

 The structure determination is technically sound.

I have read this submission. I believe that I have an appropriate level of expertise to confirm that it is of an acceptable scientific standard, however I have significant reservations, as outlined above.

# Response

**Published:** 2018-07-03

**Authors:** Igor Eliseev

**Affiliations:** St. Petersburg National Research Academic University RAS, Russian Federation

Dear Prof. Ferguson,

 We appreciate your comments on the SPR experiment, which appeared both insightful and very helpful for us.

 We first addressed the regeneration issue. The initial choice of regeneration buffer (glycine-HCl pH 2.0) was made because it efficiently eluted several anti-ErbB3 VHH fragments we were working with. We probed the receptor with a different antibody and saw no significant loss of binding after treatment with glycine buffer. However, this alternative antibody targeted a different epitope on the receptor, while the epitope of BCD090-M2 appeared very sensitive to low pH, as you suggested. We tested various regeneration conditions, and although their effect on the receptor was not as detrimental as that of glycine, we still saw some memory effects. Therefore, we finally adopted a protocol with a long dissociation phase (30 min) followed by a 10 min equilibration with running buffer, which was sufficient for complete elution of the antibody.

 Then we increased the length of the injection to 30 min to ensure that all binding curves reach equilibrium at the end of association phase. The measured binding response as a function of antibody concentration was fitted with simple 1:1 binding model by a nonlinear least-squares method which gave equilibrium constant K _D_=14 nM. We repeated the experiment twice and saw a remarkable reproducibility; the mean K _D_ was 15±1 nM. Finally, we updated the Figures 2A and 2B to present new experimental data.

# Peer review: Referee response for version 1

**DOI:** 10.5256/f1000research.14786.r30112

**Published:** 2018-03-05

**Authors:** Shi Hu

**Affiliations:** 1 Department of Biophysics, College of Basic Medical Sciences, Second Military Medical University, Shanghai, China

For the immunoglobulin-γ (IgG) antibodies, the structure usually is assembled from two identical heavy (H)-chain and two identical light (L)-chain polypeptides is well established and highly conserved in mammals. The L chain of these immunoglobulins comprises two domains, whereas the H chain folds into four domains. Currently, most of therapeutic antibodies used in clinical development are belong to such type. One exception to this conventional mammalian IgG structure is found in sera of Camelidae. Unlike the conventional heterotetrameric antibodies these sera possess special IgG antibodies. IgG antibodies, known as heavy-chain antibodies (HCAbs), are devoid of the L chain polypeptide and are unique because they lack the first constant domain (CH1). At its N-terminal region, the H chain of the homodimeric protein contains a dedicated variable domain, referred to as VHH, which serves to associate with its cognate antigen. The VHH in an HCAb is the structural and functional equivalent of the Fab fragment (antigen-binding fragment) of conventional antibodies.

 In this report, Igor et al expressed a recombinant single-domain antibody (VHH form llamas) targeting ErbB3 Protein crystallography study was used for determine the structure of VHH in different crystal forms. It is a well done crystallography report providing readers more data for the structure of VHH, and I only have a few minor suggestions:

1.  Do the authors have any information or data about the epitope of BCD090-M2?
2. Single-domain antibodies from llamas are new generation of therapeutical antibody drugs, can authors make another 3-D Alignment figure to compare BCD090-M2 with human antibodies?

I have read this submission. I believe that I have an appropriate level of expertise to confirm that it is of an acceptable scientific standard.

# Response

**Published:** 2018-07-03

**Authors:** Igor Eliseev

**Affiliations:** St. Petersburg National Research Academic University RAS, Russian Federation

Dear Dr. Shi,

We thank you for reading and commenting on our manuscript. Let us respond to your comments:

1. Initially, we hypothesized that the antibody BCD090-M2 binds to the extracellular domain III of ErbB3 (ECDIII). To test this hypothesis, we have conducted pairwise SPR binding experiments with intact ErbB3 ECD and another anti-ErbB3 antibody which targets ECDIII but saw simultaneous binding of two antibodies. Then we expressed and purified ECDIII (amino acids 329-532 of ErbB3), and also observed no interaction with BCD090-M2 in direct binding experiments. We are now performing additional experiments to determine the epitope of BCD090-M2 on the ErbB3 extracellular domain and elucidate the binding mechanism.
2. The alignment of the framework regions of our llama antibody and heavy chains of human antibodies is indeed very good. However, the CDRs of our antibody does not align well with human antibodies, e.g., those anti-ErbB3 antibodies we cited in the manuscript (target ECD I, III, II and IV). It is interesting whether llama and human antibodies recognize the same molecular features, and we certainly plan to compare our llama antibody with human anti-ErbB3 antibodies after identification of its epitope.
